# Interstitial lung disease in gefitinib-treated Japanese patients with non-small cell lung cancer – a retrospective analysis: JMTO LC03-02

**DOI:** 10.1186/1756-0500-2-157

**Published:** 2009-08-05

**Authors:** Masatsugu Nakagawa, Tsutomu Nishimura, Satoshi Teramukai, Harue Tada, Fumihiro Tanaka, Kazuhiro Yanagihara, Kiyoyuki Furuse, Hiromi Wada, Masanori Fukushima

**Affiliations:** 1 Department of Clinical Trial Design and Management, Translational Research Center, Kyoto University Hospital, Kyoto, Japan; 2 Department of Thoracic Surgery, Kyoto University, Kyoto, Japan; 3 Department of Thoracic Surgery, Hyogo College of Medicine, Nishinomiya, Japan; 4 Department of Translational Clinical Oncology, Kyoto University, Kyoto, Japan; 5 The Japan Multinational Trial Organization, Kyoto, Japan; 6 Translational Research Informatics Center, Kobe, Japan

## Abstract

**Background:**

In Japan, high incidences of interstitial lung disease (ILD) and ILD-related deaths have been reported among gefitinib-treated patients with non-small cell lung cancer (NSCLC). We investigated the efficacy of gefitinib, the incidence of ILD and risk factors for ILD in these patients.

**Findings:**

We obtained patient data retrospectively using questionnaires sent to 22 institutions. We asked for demographic and clinical data on NSCLC patients for whom gefitinib treatment had begun between July 2002 and February 2003. Data from a total of 526 patients were analyzed. The patient characteristics were as follows: 64% male, 69% with adenocarcinoma, 61% with a performance score of 0–1, and 5% with concurrent interstitial pneumonitis. The objective response proportion was 80/439 (18.2%; 95% CI: 14.7–22.0). ILD developed in 17 patients (3.2%; 95% CI 1.9–5.1%), of whom 7 died. According to multivariate analysis, female sex, history of prior chemotherapy, low absolute neutrophil count before gefitinib treatment, and adenocarcinoma histology were associated with response to gefitinib treatment. None of the factors we evaluated were associated with the development of ILD.

**Conclusion:**

The results of this study are consistent with previously published values for treatment response proportions and incidence of ILD during gefitinib treatment in Japanese patients. Future studies should be aimed at identifying factors indicating that a patient has a high probability of receiving benefit from gefitinib and a low risk of developing ILD.

## Background

In most industrialized countries, non-small cell lung cancer (NSCLC) is the leading cause of death from cancer [1]. At the time of diagnosis, the majority of NSCLC patients have advanced disease that is not amenable to curative approaches, so NSCLC is associated with a poor prognosis. Platinum-based chemotherapy provides a survival benefit of limited duration [2], and combinations of newer active agents (such as gemcitabine and paclitaxel) with platinum have produced further improvement in survival [3,4]. Docetaxel has been approved as a second-line chemotherapy agent on the basis of randomized trials involving patients in whom first-line chemotherapy was unsuccessful, but the objective response proportions to docetaxel are only 5–10%, and treatment is associated with only a modest survival benefit [5,6].

Gefitinib is an orally active selective inhibitor of the epidermal growth factor receptor (EGFR) tyrosine kinase and has been found to have antitumor activity in patients with advanced NSCLC who have undergone prior treatment. According to several multicenter phase I trials [7-9], diarrhea, skin rashes, and nausea are common adverse events associated with gefitinib treatment, but most of these adverse events are mild. Based on the results of two large-scale multicenter phase II studies (IDEAL 1 and 2; Iressa Dose Evaluation in Advanced Lung Cancer) [10,11], in July 2002 gefitinib was approved in Japan for the treatment of inoperable or recurrent NSCLC. However, since approval, a number of deaths due to gefitinib-induced interstitial lung disease (ILD) have been reported [12,13], and the high incidences of ILD and associated mortality have become a matter of great concern in Japan. Risk-benefit assessments have become critical in the use of gefitinib. We report here the results of a retrospective study conducted by the Japan Multinational Trial Organization (JMTO: ) to investigate the response proportion, factors predicting response, and the incidence of and risk factors for ILD in gefitinib-treated NSCLC patients.

## Methods

Patients with histologically or cytologically confirmed NSCLC of any clinical or pathological stage, who began taking gefitinib between July 2002 and February 2003, were eligible for inclusion in the study. We obtained patient data using questionnaires sent to 22 institutions, and retrospectively analyzed the information provided in the returned questionnaires, including laboratory findings. The study protocol (JMTO LC03-02) was approved by the ethics committees of JMTO and the participating institutions.

Baseline assessments included age, sex, histological type of NSCLC, clinical or pathological stage of cancer at diagnosis (using the current TNM classification system [14]), Eastern Cooperative Oncology Group (ECOG) performance status (PS), history of smoking before starting gefitinib treatment, medical history (including prior anticancer therapy), concurrent pulmonary disease (e.g. interstitial pneumonitis or pulmonary emphysema), and laboratory findings (complete blood cell count, blood biochemistry, arterial blood gas analysis data). Diagnoses of concurrent pulmonary disease were made by each institution on a clinical basis.

The endpoints of this study were response to gefitinib treatment or occurrence of a serious adverse event during gefitinib treatment or death due to the serious adverse event. Adverse events were evaluated using the National Cancer Institute Common Toxicity Criteria (NCI-CTC), version 2.0, and a serious adverse event was defined as being an NCI-CTC grade 4 adverse event, or any experience that resulted in death, was life-threatening, required inpatient hospitalization or prolongation of existing hospitalization, resulted in persistent or significant disability/incapacity, or caused a congenital anomaly/birth defect, according to the definition published by the International Conference on Harmonisation of Technical Requirements for Registration of Pharmaceuticals for Human Use [15]. In this study, ILD was assumed to be interstitial pneumonitis as diagnosed by each institution on a clinical basis.

If patients had measurable disease, tumor response was assessed on the basis of medical records, and if not mentioned in the medical records, the Response Evaluation Criteria in Solid Tumors (RECIST) [16] or the World Health Organization (WHO) criteria [17] were used for assessment. The objective response was calculated as the proportion of patients with a complete or partial response.

Associations between serious adverse events or objective response and patient characteristics were assessed using a logistic regression model. Odds ratios (ORs) and 95% confidence intervals (CIs) were estimated using univariate analysis, and a backward elimination procedure was used to identify significant predictors in the multivariate analysis. Because of the comparability of the ORs, the units used to measure absolute neutrophil count and lactate dehydrogenase (LDH) level were Log transformed. Predictive response probabilities were estimated based on a final multivariate logistic regression model for evaluating associations between response and ILD occurrence. All statistical analyses were carried out using SAS version 8.02 (SAS Institute, Cary NC, USA).

## Results

### Patient characteristics

A total of 536 patients were enrolled in this study, of which 526 patients were eligible for analysis (10 patients were excluded from analysis because they began taking gefitinib before July 2002 or after February 2003). The patient characteristics and laboratory findings are summarized in Table 1.

**Table 1:** Patient characteristics (n = 526).

Characteristic	No. patients	% patients	Mean (± SD)
Age (years)	526		66.2 (range 27–91)

Sex			
Male	336	64	
Female	190	36	

Histological type			
Adenocarcinoma	360	69	
Squamous cell carcinoma	123	23	
Other	43	8	

History of smoking			
No	170	32	
Yes	319	61	
Unknown	37	7	

ECOG performance status			
0–1	321	61	
2–4	191	36	
Unknown	14	3	

Concurrent interstitial pneumonitis			
No	500	95	
Yes	26	5	

Concurrent pulmonary emphysema			
No	447	85	
Yes	77	15	
Unknown	2	<1	

History of pulmonary tuberculosis			
No	491	93	
Yes	34	7	
Unknown	1	<1	

Prior surgery for lung cancer			
No	337	64	
Yes	189	36	

Prior chemotherapy for lung cancer			
No	115	22	
Yes	411	78	

Prior thoracic radiotherapy for primary lung cancer			
No	330	63	
Yes	194	37	
Unknown	2	<1	

Concurrent chemotherapy with agent other than gefitinib			
No	485	92	
Yes	41	8	

Thoracic radiotherapy concurrent with or after gefitinib treatment			
No	478	91	
Yes	48	9	

Absolute neutrophil count (/μL)	485		4786 (± 3331)
Eosinophil count (/μL)	483		163 (± 181)
Creatinine level (mg/dL)	496		0.77 (± 0.44)
Platelet count (×10^4^/μL)	515		25.8 (± 10.3)
Albumin level (g/dL)	424		3.67 (± 0.58)
Aspartate aminotransferase level (IU/L)	487		25 (± 20)
Alanine aminotransferase level (IU/L)	515		23 (± 25)
Lactate dehydrogenase level (IU/L)	495		279 (± 318)

The mean patient age was 66.2 years (range 27–91 years). A total of 336 patients (64%) were male, 360 patients (69%) had adenocarcinoma, and 319 patients (61%) were current or former smokers. In 26 patients (4.9%), NSCLC was accompanied by concurrent interstitial pneumonitis. Gefitinib was given as a first-line chemotherapeutic agent in 115 patients.

With respect to the laboratory findings before gefitinib treatment, absolute neutrophil count, aspartate aminotransferase (AST) level, alanine aminotransferase (ALT) level, and LDH level were reported for 485, 487, 515, and 495 patients, respectively (Table 1).

### Response to treatment

For 439 patients (83%), tumor response was reported and evaluable. Complete and partial responses were observed for 7 and 73 patients, respectively. The objective response proportion was 80/439 (18.2%; 95% CI: 14.7–22.0), and the disease control proportion, which was the objective response plus the proportion of patients with stable disease, was 237/439 (54.0%).

### Factors predicting tumor response

The association between tumor response and various clinical factors was assessed using logistic regression. In the univariate analysis (Table 2), sex, histological type, history of smoking, concurrent pulmonary emphysema, history of prior chemotherapy, high absolute neutrophil count, low LDH level, and high serum albumin level were found to be associated with tumor response (p < 0.1). In the multivariate analysis (Table 3), female sex (OR: 4.35; 95% CI: 2.40–7.90; p < 0.001), history of prior chemotherapy (OR: 0.34; 95% CI: 0.18–0.65; p = 0.001), high absolute neutrophil count (OR: 0.85 per 1000/μL; 95% CI: 0.75–0.93; p = 0.017), and adenocarcinoma histology (OR: 2.35; 95% CI: 1.03–5.35; p = 0.043) were statistically significantly associated with tumor response.

**Table 2:** Predictive factors for tumor response and risk factors associated with ILD (univariate analysis)

Factor	Response proportion (%)	Odds ratio (95% CI)	p-value	Incidence of ILD (%)	Odds ratio (95% CI)	p-value
Age	-	1.01 (0.98–1.03)	0.644		1.00 (0.95–1.04)	0.823

Sex						
Male	8.4	1	-	2.7	1	-
Female	34.3	5.68 (3.33–9.69)	<0.001	4.2	1.60 (0.61–4.21)	0.344

Histological type						
Non-adenocarcinoma	6.0	1	-	3.6	1	-
Adenocarcinoma	23.6	4.86 (2.27–10.42)	<0.001	3.1	0.84 (0.31–2.33)	0.737

History of smoking						
Yes	10.5	1	-	3.1	1	-
No	33.8	4.33 (2.57–7.29)	<0.001	4.1	1.33 (0.50–3.57)	0.573

ECOG performance status						
2–4	14.4	1	-	2.6	1	-
0–1	20.3	1.51 (0.89–2.59)	0.129	3.6	1.39 (0.48–4.00)	0.549

Concurrent interstitial pneumonitis						
No	18.9	1	-	3.2	1	
Yes	5.0	0.23 (0.03–1.72)	0.151	3.8	1.21 (0.15–9.49)	

Concurrent pulmonary emphysema						
No	19.9	1	-	3.1	1	
Yes	7.1	0.31 (0.11–0.88)	0.028	3.9	1.25 (0.35–4.47)	

Prior surgery						
No	16.0	1	-	3.6	1	-
Yes	22.2	1.49 (0.91–2.44)	0.111	2.6	0.74 (0.26 -2.12)	0.571

Prior chemotherapy						
No	28.6	1	-	2.6	1	-
Yes	15.5	0.46 (0.27–0.79)	0.005	3.4	1.32 (0.37–4.66)	0.670

Prior thoracic radiotherapy						
No	19.3	1	-	3.9	1	-
Yes	16.6	0.83 (0.50–1.38)	0.468	2.1	0.51 (0.17–1.60)	0.250

Concurrent chemotherapy with agent other than gefitinib						
No	17.3	1	-	3.5	-	-
Yes	28.6	1.91 (0.88–4.15)	0.103	0	-	-

Thoracic radiotherapy concurrent with or after gefitinib treatment						
No	18.8	1	-	3.1	1	-
Yes	12.8	0.64 (0.24–1.69)	0.364	4.2	1.34 (0.30–6.05)	0.702

Absolute neutrophil count *	-	0.84 (0.75–0.95)	0.004	-	0.96 (0.81–1.14)	0.609
Aspartate aminotransferase level **	-	1.00 (0.99–1.01)	0.967	-	0.99 (0.95–1.03)	0.635
Lactate dehydrogenase level ***	-	0.82 (0.65–1.03)	0.085	-	0.95 (0.71–1.27)	0.724
Albumin level ****	-	1.54 (0.97–2.44)	0.067	-	1.65 (0.67–4.07)	0.278

* per: 1000/μL **: per IU/L ***: per 100 IU/L ****: per g/dL

**Table 3:** Factors predicting tumor response (multivariate analysis)

Factor	No. patients	Odds ratio (95% CI)	p-value
Sex			
Male	256	1	-
Female	152	4.35 (2.40–7.90)	<0.001
Prior chemotherapy			
No	82	1	-
Yes	326	0.34 (0.18–0.65)	0.001
Absolute neutrophil count	408	0.85 (0.75–0.93)*	0.017
Histological type			
Non-adenocarcinoma	127	1	-
Adenocarcinoma	281	2.35 (1.03–5.35)	0.043

Valuables with p < 0.05 were selected using a backward elimination procedure.

*per 1000/μL.

### ILD and other serious adverse events

Serious adverse events occurred during gefitinib treatment in 66 of 526 patients. ILD developed in 17 patients (3.2%; 95% CI: 1.9%–5.1%), 7 (41%) of whom died during treatment. Hepatic dysfunction occurred in seven patients, one of whom died during treatment. Other serious adverse events included skin rash, diarrhea, aspiration pneumonia, perforative peritonitis, acute enteritis, convulsion, epilepsy, cancerous pain.

### Analysis of factors affecting ILD

The association between ILD and various clinical factors was evaluated using logistic regression. In the univariate analysis, none of the factors we evaluated were found to be associated with the development of ILD, including sex, history of smoking, concurrent interstitial pneumonitis, history of prior chemotherapy, or poor ECOG PS.

### Analysis of the relation between response and ILD

**Figure 1:**
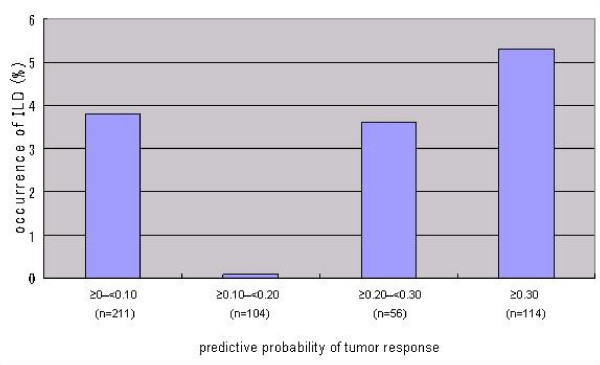
**Relationship between predictive probability of tumor response and ILD**.

We assessed the association between response and ILD occurrence. When the predictive probability of a response was estimated based on related factors (sex, history of prior chemotherapy, absolute neutrophil count, and histology) for each patient, no association between response probability and occurrence of ILD was detected (chi-square test, p = 0.379) (Figure 1). When the probability of a response was 0–0.10, the incidence of ILD was 3.8% (8/211, 95% CI: 1.7%–7.3%), and when the probability was above 0.30, the incidence was 5.3% (6/114, 95% CI: 2.0%–11.1%).

## Discussion

In the present study, the gefitinib response proportion was 18.2%, which is similar to values determined in other studies and clinical trials [10,18,19]. The response proportion among patients with a history of prior chemotherapy was 15.5%, which was somewhat higher than values observed in clinical trials of docetaxel chemotherapy for previously treated NSCLC patients (10.8% and 7.1%) [5,6].

In this study, female sex, no history of prior chemotherapy, low absolute neutrophil count, and adenocarcinoma histology were associated with tumor response. There have been a number of prior studies of the predictive factors for tumor response in gefitinib-treated NSCLC patients in Japan [20-24], and results of these are summarized in Table 4. Although female sex and adenocarcinoma histology have also been found to be associated with tumor response in other studies, absence of prior chemotherapy and low absolute neutrophil have not. The reason for the differences between the previous and the present results is unknown, but prior chemotherapy treatment may adversely affect tumor response to gefitinib.

**Table 4:** Risk factors for ILD associated with gefitinib use and predictive factors for tumor response in Japanese patients as determined by studies in the literature

**Reference**	**No. patients**	**No. cases ILD (%)**	**No. ILD deaths (%)**	**Risk factors for ILD**	**Response Proportion (%)**	**Predictive factors for tumor response**
National Cancer Center, Japan (2004) [20]	112	6 (5.4)	4 (3.6)	-Pre-existing pulmonary fibrosis	33	-No history of smoking -No history of thoracic radiotherapy

Okayama Lung Cancer Study Group (2005) [21]	330	15 (4.5)	8 (2.4)	-PS 2–4 -Pre-existing pulmonary fibrosis -Prior thoracic irradiation	22	Not investigated

AstraZeneca (2004) [22]	3322	193 (5.8)	83 (2.5)	-PS 2–4 -History of smoking -Coexisting IP -Prior chemotherapy	7.8	Not investigated

West Japan Thoracic Oncology Group (2006) [23]	1976	70 (3.5)	31 (1.6)	-History of smoking -Male -Coexisting IP	17.6	-PS 0–1 -No history of smoking -Female -Adenocarcinoma -Metastatic disease

AstraZeneca (2007) [24]	1482	59 (4.0)	ILD-related deaths In patients with ILD were 31.6%	-PS 2–4 -History of smoking -Age >55 years -Recent NSCLC diagnosis, -Reduced normal lung on computed tomography scan -Preexisting chronic ILD -Concurrent cardiac disease	Not investigated	Not investigated

Present study	526	17 (3.2)	7 (1.3)	No factors found to be significant	18.2	-Female -No previous chemotherapy -Low absolute neutrophil count -Adenocarcinoma

The incidence of ILD in this study was 3.2%, which is similar to the values determined in other studies (3.5–5.8%) (Table 4). Determining the incidence of anti-EGFR therapy-related ILD in NSCLC is complicated by many factors, including the underlying neoplastic disease, adverse events caused by other chemotherapy agents, oxygen treatment, radiation therapy, and opportunistic infections. In a study conducted by the West Japan Thoracic Oncology Group (WJTOG) [23], the incidence of ILD in gefitinib-treated NSCLC patients was found to be 3.5%. In that study, ILD was defined as diffuse interstitial changes or ground-glass appearance, and the diagnosis was confirmed on the basis of radiological findings and clinical data by a central review committee comprising chest radiologists, chest physicians, and oncologists. In the present study, ILD was defined as a diagnosis of interstitial pneumonitis during gefitinib treatment, as diagnosed by each institution on a clinical basis. A central review committee was not involved. Despite this methodological difference, the incidence of ILD in the present study population was similar to the value found in the WJTOG study.

Risk factors for ILD have been identified in a number of studies of Japanese gefitinib-treated NSCLC patients (Table 4). In the present study, we also attempted to identify risk factors for ILD in gefitinib-treated NSCLC patients, but unlike the previous studies, none of the factors we evaluated were found to be significant, including sex, history of smoking, concurrent interstitial pneumonitis, history of prior chemotherapy, and poor PS. We cannot explain why this might be, but other risk factors that we did not investigate may possibly be associated with ILD.

In the interests of patient welfare, we need criteria by which patients with factors predicting tumor response or survival benefit and without factors predisposing to ILD can be selected. In the WJTOG study it was concluded that patient selection on the basis of female sex and the absence of a history of smoking will increase the clinical benefit of treatment with gefitinib and reduce the risk of ILD [23]. In the present study, we found no factors that clearly increase the benefit or decrease the risk. We did not find an association between the predictive response probability and the occurrence of ILD. The mechanism by which gefitinib might cause ILD is unclear, but that may be because the site at which gefitinib acts is different from that at which ILD manifests. Because of the considerable risk to patients, further studies investigating risk factors are warranted. The limitations of the present study were the retrospective design, the relatively small number of patients, and the conduction of the study using questionnaire, but the results of present study were similar to those of the previous reports.

## Conclusion

The results of this study are consistent with previously published values for treatment response proportions and incidence of ILD during gefitinib treatment. In the interests of patient welfare, ILD in gefitinib-treated NSCLC patients should be investigated further in large-scale prospective studies, in which patients should be selected on the basis of factors affecting ILD and predicting tumor response.

## Competing interests

This study was supported by AstraZeneca.

## Authors' contributions

MN participated in the design of the study, carried out the study, and drafted the manuscript. TN carried out data management and helped to draft the manuscript. ST participated in the design of the study and performed the statistical analysis. HT participated in the design of the study and carried out data management. FT participated in the design of the study and carried out the study. KY, KF and HW participated in the design of the study and carried out the study. MF conceived the study, participated in its design and coordination, and helped to draft the manuscript. All authors read and approved the final manuscript.
